# Stranded assets in European agriculture during food system transformations

**DOI:** 10.1038/s43016-025-01283-z

**Published:** 2026-01-19

**Authors:** Anniek J. Kortleve, José M. Mogollón, Helen Harwatt, Martin Bruckner, Baoxiao Liu, Paul Behrens

**Affiliations:** 1 Institute of Environmental Sciences (CML), Leiden University, Leiden, the Netherlands; 2 Chatham House, The Royal Institute of International Affairs, London, UK; 3 Oxford Martin School, University of Oxford, Oxford, UK; 4 Institute for Ecological Economics, University of Economics and Business, Vienna, Austria

**Keywords:** Climate-change policy, Environmental economics

## Abstract

Dietary shifts, particularly reduced animal-sourced food (ASF) consumption in high-income countries, risk stranding substantial ASF-related assets. Linking agricultural and economic data to global multi-regional input–output models, we show that ASF assets represent 78% of EU27 + UK fixed agricultural assets, with €158 billion linked to livestock and €100 billion to feed production. We estimate that ASF reductions in EU27 + UK consumption of 9.5%, 60% and 100% could strand 18%, 50% and 77% of these assets, respectively. Current depreciation rates suggest there is generally sufficient time to phase out assets, offering pathways to limit stranding. Policy- and climate-induced stranding risks are intertwined and should both be incorporated into financial modelling as overlapping transition pressures. Given food producers’ high exposure to stranding risks cascade throughout supply chains, integrated policy support to repurpose or phase out ASF-related assets is essential to avoid delays in sustainable food system transformations.

## Abstract (web summary)

Stranded assets could pose a challenge to food system transformation. Estimates of the stranded agricultural assets that may arise from a shift to plant-based diets in the European Union and UK underscore the need to refocus support mechanisms for ensuring a just transition.

## Main

The global food system accounts for between one-quarter and one-third of all anthropogenic greenhouse gas emissions and is a major contributor to deforestation, biodiversity loss and water pollution^1–3^. Recent studies show that food system emissions alone are sufficient to breach the 1.5 °C and perhaps even the 2 °C warming thresholds^4,5^. This finding underscores the urgent need for a major food system transformation, involving dietary change, reductions in food waste and improvements in food production^4,6^.

Among these approaches, shifting to plant-rich diets offers the largest greenhouse gas reduction potential in higher-income regions^3,7^. Transitioning away from animal agriculture would entail a large-scale reorganization of the food system, with increased investments in legume cultivation, horticulture and alternative proteins, whereas some assets in meat and dairy could risk being stranded^8,9^. Here we define stranded assets as unanticipated or premature write-offs of fixed assets^10^.

Research on stranded assets has largely focussed on fossil fuel infrastructure, where stranded assets could reach US$1 trillion, with most losses occurring in OECD (Organisation for Economic Co-operation and Development) countries^11–13^. The scale of these potentially stranded assets often leads vested interests to resist climate policy and energy transitions^14^. Although asset stranding is anticipated across the fossil fuel industry under scenarios consistent with meeting climate targets^11–13,15^, agricultural stranding risks are likely to vary by sub-sectors, practices or regions^16^. The overall magnitude of future stranded assets will depend on a range of factors including climate impacts, biodiversity loss, extreme weather events, land degradation, resource scarcity and the proactive or reactive responses of governments, food producers and consumers^8–10,16,17^.

These responses, including dietary change, represent transition risks that can lead to asset stranding, while climate impacts represent physical risks. This study focuses on stranding driven by transition risks, represented as exogenous dietary scenarios rather than policy-specific interventions or endogenous market dynamics. Whereas macroeconomic studies have highlighted the broad economic costs of food system transitions (for example, ref. ^18^), others have argued that proactively addressing stranded assets may be essential for unlocking the policy window through which rapid transformation can occur^19^.

Stranded assets pose several challenges across society. For businesses, they represent investments that are difficult to sell or convert back into cash (low liquidity) at risk of sudden loss in economic or functional value. Governments may face market failures from unregulated externalities, while at a macroeconomic level, prolonging the use of unsustainable assets could harm productivity, economic growth, social welfare and public finances^10^. The heavy financialization of the European Union and UK food system has heightened vulnerabilities and exposure to asset stranding, especially through an over-reliance on high-carbon-emitting investments^8^. The resulting stranded assets are often illiquid and at risk of devaluation, with potential ripple effects across supply chains^9^.

In this study, we explore the potential for asset stranding under different levels of ASF substitution with plant-based foods. We model reductions of 9.5%, 60% and 100% in ASF consumption from current levels, based on ranges determined in the EAT–*Lancet* diet (representing moderate, low and zero ASF scenarios; see Methods for details). We assess the potential stranding of fixed assets^10^, including farm buildings, machinery, equipment and breeding livestock. Land assets are analysed separately due to their greater potential for repurposing and the complexities of valuing future land use. These include distortions from subsidies, tax rules and commodity prices^8^, and the exclusion of ecosystem services, such as biodiversity, water regulation and carbon sequestration, from market prices^10^. Here we focus on the changes in asset values under different dietary transitions.

## Results

### Farm-level asset landscape

**Fig. 1:**
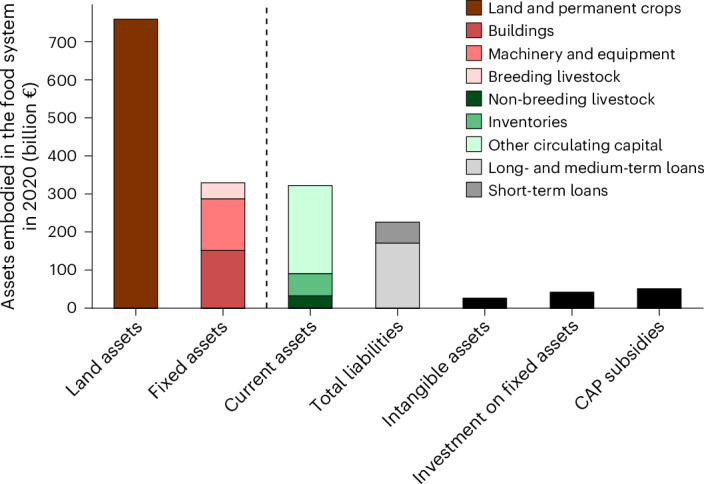
Overview of asset types within the EU27 + UK food system in 2020. Land assets include land value and permanent crops (for example, orchards, vineyards). Fixed assets are divided into buildings, machinery and equipment and breeding livestock. Current assets include non-breeding livestock, inventories (stocks of products owned by the farm for input use or sale, whether produced or purchased) and other current assets (cash, business receivables and assets easily sold or payable within a year). Total liabilities, representing farm debt, include short-term and long- to medium-term loans. Intangible assets are either tradable (quotas, rights) or non-tradable (software, licences). CAP subsidies include the full CAP budget for the EU27 + UK. Assets within the full agricultural system (food and non-food use) are shown in Supplementary Fig. 2. Source data

In 2020, the combined value of farm-level land and fixed assets used for food production in the EU27 + UK totalled €1.1 trillion. These food system assets were distributed as follows: land and permanent crops (70%); buildings (14%); machinery and equipment (12%); and breeding livestock (4%) (Fig. 1). In addition to these long-term assets, the food system includes faster-circulating assets or current assets (that is, those used within a single operating cycle, typically one year) comprising non-breeding livestock, inventories, and other circulating capital, valued at €322 billion (Fig. 1). Total liabilities of €226 billion are excluded from fixed assets, being linked to highly liquid markets that enable risk movement^10^. Yearly investments in fixed assets total €42 billion and intangible assets equate to €26 billion (Fig. 1). For reference, food-related subsidies from the EU’s Common Agricultural Policy (CAP) totalled €51 billion (Fig. 1).

### Asset stranding under dietary transitions

To explore potential stranded assets under a dietary transition, we link assets to specific products, covering all animal- and plant-sourced products (that is, animal feed) required to produce ASFs. We also split assets for producing plant-based foods for direct human consumption by asset type. Overall, 78% of the fixed assets embodied in the food system are linked to ASFs, with €158 billion allocated to livestock production and €100 billion to upstream animal feed production. It is important to highlight that approximately 40% of stranded assets from a plant-based shift would be in crop agriculture for feed, implying the need for distinguishing policies for both animal- and plant-agriculture.

**Fig. 2:**
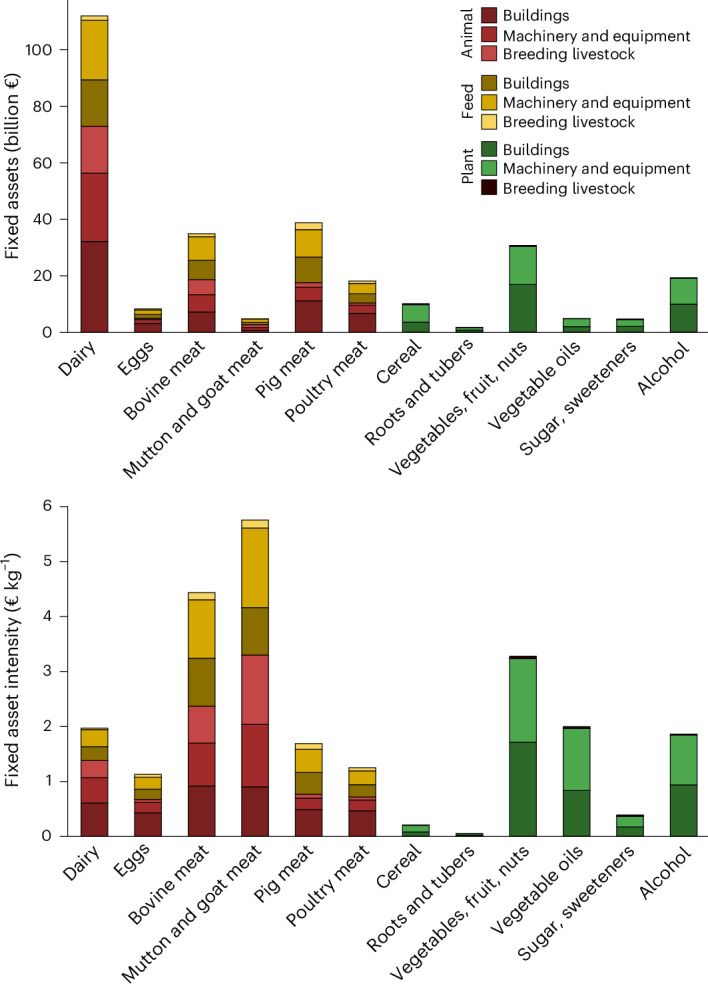
Allocation of animal, feed and plant assets across food production by asset type. The upper panel shows total fixed assets for the EU27 + UK in 2020, and the lower depicts average asset intensity (asset value per unit of food produced). Source data

Among ASF assets, EU27 + UK dairy leads with €109 billion (€71 billion in livestock and €38 billion in feed production; Fig. 2), with 16% embodied in breeding livestock, 40% in machinery and equipment and 44% in buildings. Feed assets are highest in the EU27 + UK dairy value chain (39% of feed assets), followed by pig meat (21%) and bovine meat (16%) (Fig. 2). Asset intensity, or the asset value per unit of food produced, is highest in beef, lamb and goat meat products (Fig. 2).

**Fig. 3:**
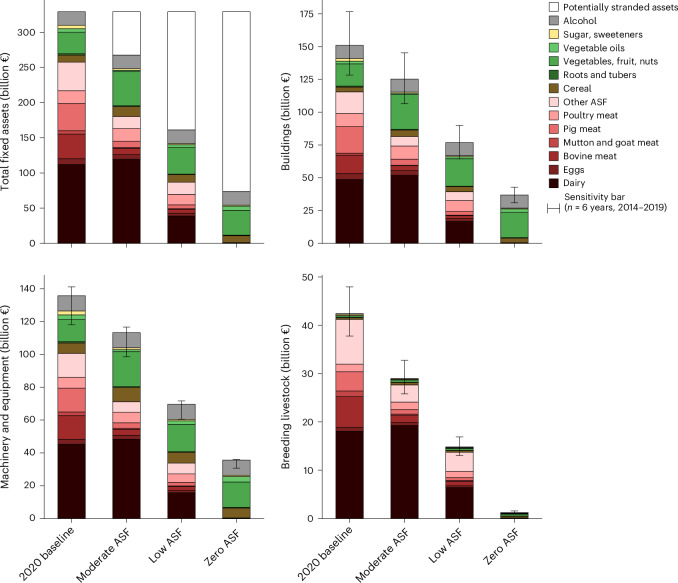
Embodied fixed assets under EAT–*Lancet* dietary scenarios. ‘Other ASF’ includes animal fats, offal and other meat. Range markers represent sensitivity intervals of asset values (*n* = 6 years, 2014–2019; Methods provide details, and Supplementary Fig. 5 provides the mean deviation of each farm type–region combination). Source data

A 9.5% reduction in ASF consumption in the EU27 + UK (moderate ASF scenario) potentially strands €61 billion of fixed assets (or 20% of the total); a 60% reduction (low ASF scenario) €168 billion (49%) and a 100% reduction (zero ASF scenario) €255 billion (73%) (Fig. 3). The steepest declines are in breeding livestock assets, with reductions of 31%, 67% and 98% in the moderate, low and zero ASF, respectively (Fig. 3). Buildings and machinery and equipment asset classes follow a similar trajectory, showing a 16–17%, 49% and 73–75% decline for each scenario, respectively (Fig. 3). For zero ASF, some breeding livestock assets remain, reflecting niche, multifunctional roles of farm animals in crop production (Fig. 3).

Land assets could see decreases of 19%, 48% or 71% under the moderate ASF, low ASF or zero ASF scenarios. This implies a land asset value reduction of €153 billion, €370 billion or €551 billion. The sharpest declines occur in bovine and pig meat in the moderate ASF scenario; under the low and zero ASF scenarios, there is a steep decline in the dairy value chain. To maintain land asset values under these shifts, the average value of land across all agricultural uses in the EU + UK would need to reach approximately €5,000 ha^−1^ in 2020, potentially supported by revenues from environmental services, plant-based food or other economic activities. For context, the same land currently receives approximately €300 ha^−1^ yr^−1^ of CAP support.

### Plant-based asset growth and repurposing

The decline in ASF assets is slightly offset by an increase in plant-based assets, following more plant-based food consumption (Supplementary Fig. 1). This results in a 3–24% rise in buildings and a 0.5–20% rise in machinery and equipment assets, with the largest increases seen in vegetables, fruit and nuts (Fig. 3). Our analysis does not address whether these assets are newly developed or repurposed from ASF production systems. Future innovation in plant-based production, such as precision fermentation and vertical farming, may further influence the asset intensity of plant-based production.

### Depreciation and timelines for asset phase-out

Non-land assets depreciate over time. These assets could be phased out following potential dietary transitions^17^. The annual depreciation applied in agriculture is generally similar or higher than in fossil infrastructure^15^. Using the current annual depreciation rates of 9% (ref. ^20^), all redundant ASF assets under the low ASF scenario would depreciate fully within 10 years, whereas a complete phase-out under the zero ASF scenario would require approximately 30 years (Supplementary Fig. 3). This suggests that a systematic phase-out of ASF assets accompanied by a complete investment stop, would leave minimal residual value and limit the extent of stranded assets. However, an accelerated phase-out by 2030 could result in €99 billion stranded assets (Supplementary Fig. 3). Evaluating trade-offs between different policy-led transition timelines is critical for understanding the potential economic impact of managed transitions compared to a forced phase-out by the climate crisis.

Depreciation is used here as an accounting proxy to estimate the potential pace of asset phase-out under managed transition scenarios. However, this approach does not account for real-world barriers to phase-out, such as subsidies, tax regimes or financial constraints that may delay disinvestment in ASF assets. Even fully depreciated assets may remain operational if they continue to generate revenue, which can delay the start of phase-out in the absence of stronger transition policies.

## Discussion

### Policy responses to stranded asset risks

In any of these scenarios, targeted policy intervention is essential, including support for debt and asset depreciation management, and general transition assistance for farmers. A socially just transition that addresses existing social and economic inequalities and vulnerabilities within the food system is crucial to ensure that transition costs and benefits are equitably distributed, ultimately fostering greater public support for the transition^21–23^.

While the general need for a well-managed transition is widely acknowledged^23^, the quantification of stranded asset risks, €61 billion, €168 billion and €255 billion under the moderate, low and zero ASF scenarios, adds crucial specificity for policy design. By translating abstract transition risks into concrete financial terms, such figures can guide the scale and timing of depreciation support, inform compensation schemes and support realistic regulatory timelines. Our analysis also provides asset intensity per kilogram of food for different food items (Fig. 2), offering a basis for tailoring compensation and support mechanisms according to food-level exposure. Similar approaches are already used in the energy sector, where compensation for stranded coal assets or displaced workers is estimated per MW or per worker to support labour reskilling and regional diversification^24–26^.

The EU’s Just Transition Mechanism, established under the Green Deal, allocates ~€55 billion to support social and economic adjustments in high-carbon regions^27^. Although the mechanism does not currently cover agriculture, the European Economic and Social Committee has called for a dedicated just transition framework for the agrifood sector, an idea under consideration in the CAP post-2027 reform^28^. Without targeted support, high stranded asset exposure, especially in bovine, pig meat and dairy systems, may delay EU dietary and climate action by increasing political resistance or financial vulnerability among producers^19^. Our analysis helps identify where such friction is likely and the scale of the issue that policy levers, such as aligning depreciation schedules, reforming agricultural subsidies or providing liquidity support, can help address, reduce resistance and enable more rapid transformation.

Whereas some asset stranding may be unavoidable under a transition away from ASFs and in response to the climate crisis, the key policy challenge lies in determining when, where and how these costs arise, along with, importantly, how they are distributed. At the farm level, stranded assets pose direct financial risks to farmers, particularly those with high sunk costs or long investment cycles and limited liquidity, who may face loan defaults or business failure if transition support is inadequate^10^. Financial risk is further amplified by short-term decision-making incentives and path dependency, leaving farmers with few viable exit strategies without public support^10^. When such constraints are widespread across Europe, they inhibit the reallocation of land, labour and capital towards plant-based production systems, creating system-level delays even when such pathways are technically and economically feasible.

In this context, CAP reforms are critical, as current agricultural subsidies may inadvertently contribute to stranded assets by incentivizing investment in specific practices or crops that are not aligned with environmental priorities, evolving market demands and climate risks^29^. For example, livestock-specific subsidies encourage investments that risk becoming stranded if consumer preferences shift towards more plant-rich diets or if climate change makes livestock production economically inviable^8,10^. Redirecting CAP budgets away from ASFs and towards repurposing support, liquidity assistance and crop production is one of the clearest policy levers to weaken existing ASF lock-ins, which are system-wide but may materialize through farm-level decisions, and to reduce transition delays.

Assets may be repurposed for alternative uses, with emerging opportunities in plant-based agriculture, such as precision farming, alternative protein production and regenerative farming. Examples include converting chicken sheds, dairy barns and pig barns into facilities for growing mushrooms, hemp, microgreens and specialty vegetables and herbs^30,31^. Besides the building structure itself, existing infrastructure such as cooling cells, feeders, watering systems and computer systems, can often be repurposed to support greenhouse operations^30^. In addition, retrofitting infrastructure beyond the farm gate, particularly in the manufacturing sector, presents a capital-efficient strategy for rapidly scaling up production capacity for plant-based proteins^32^. There may also be opportunities for repurposing assets for other sectors, especially with respect to buildings, energy generation, tourism and more. Whereas our results show that potential ASF-related losses far outweigh gains in plant-based assets, we do not assess whether these gains reflect new or repurposed assets. Further research can target the effects of partial offsets when evaluating transition costs and mitigation opportunities at the farm level.

From a climate perspective, failure to transition away from ASF production and consumption could exacerbate asset stranding risks as climate impacts on agriculture intensify. Both a faster decarbonization and more severe impacts of climate change could drive higher levels of asset stranding, increasing the chances of economic, social and political repercussions^16^. Additionally, low-animal welfare practices, combined with climate risks, may increase the likelihood of zoonotic and epizootic events within livestock populations^33^. Whereas stronger regulatory responses to animal welfare and biosecurity concerns could help mitigate disease risks, they would also accelerate asset stranding, especially in intensive, high-risk animal agriculture systems^34^.

Despite uncertainties surrounding transition pathways, the inertia of the climate system guarantees that even if greenhouse gas emissions were halted immediately, the risks of asset stranding in the food system would continue to grow^16^. Climate change is probably already contributing to agricultural asset stranding by driving extreme weather, altering water supplies and negatively impacting crop yields and the growth of dairy, meat and fish stocks^16,17^. Adaptive food governance is therefore essential, including diversification of agricultural production, investment in sustainable farming practices and transition support for farmers adapting to new market conditions^8^. However, while such strategies can mitigate some physical risks from climate change, they are unlikely to address all potential sources of asset stranding^16^.

Meanwhile, investors currently favour on-farm climate solutions, such as regenerative agriculture and feed additives, over demand-side measures such as promoting plant-based diets^35^. This emphasizes the need for policy interventions that encourage transitions towards more plant-based diets, for instance, through measures supporting livestock reductions^36^ and promoting plant-based alternatives^37,38^. Given the uncertainties surrounding the efficacy of on-farm livestock solutions and their limited capacity to address broader environmental harms^3,39^, investors should take a more proactive role. Rather than viewing at-risk assets solely as financial exposure to be managed, they must support the deliberate phase-out of a large proportion of ASF infrastructure through transition finance, helping to avoid prolonged lock-in and enabling a more rapid food system transformation^19^.

### Systemic and downstream repercussions

The interconnected nature of the food system, characterized by strong investment synergies across different asset types, means that stranding can propagate through supply chains^10^. The stranding of physical assets such as farm buildings, irrigation systems and crop fields can have cascading impacts across food supply chains, affecting other assets such as business networks and cooperatives reliant on consistent agricultural production. These disruptions may destabilize local communities and erode intangible assets such as knowledge, social capital and place-based expertise, elements often undervalued in financial accounting, but difficult to restore once lost^10^. This underscores the need for responses that go beyond financial risk management but that also consider the broader social impacts of asset stranding^16^.

The effects of stranded agricultural assets extend beyond primary production, leading to cascading impacts across multiple sectors^8,17^. For example, food processing facilities producing animal by-products such as leather and casein may experience supply constraints^38^, whereas logistics companies could face underutilization of refrigerated trucks and live animal transport infrastructure. Retailers may need to repurpose meat-focussed display areas and ASF-focussed financial institutes could see declines in the collateral value of loans tied to livestock assets. Those in the pharmaceutical industry that are heavily reliant on animal agriculture for antibiotic sales^40^, would experience reduced demand, affecting upstream supply chains, research and investments. In regions where tourism is closely linked to animal agriculture, revenue losses could result in additional asset stranding.

As the food system transitions towards plant-sourced foods, assets will shift, but their location, concentration and size will be naturally very different, creating new vulnerabilities and opportunities. Our stranded asset calculations may underestimate these broader, cascading risks in infrastructure beyond direct food production, such as transportation, storage facilities, electrification and other on-farm resources. It also does not account for asset stranding outside the European Union and UK, even though global markets are deeply interconnected.

Farmers remain particularly vulnerable in this transition due to their limited profitability across many areas of the EU + UK and high degree of lock-in from long-term investments^22,41^. In a food system where economic power is largely concentrated among manufacturers and retailers, farmers’ capacity to adapt to dietary shifts is restricted. This reinforces the need for broad governmental action to reorganize support mechanisms and ensure a just transition through targeted agricultural policies^21–23,38,41^, informed by quantified stranded asset risks in the livestock and feed systems.

## Methods

Agricultural assets are commonly classified into natural, physical, financial, human and social assets. In this study, we assess the potential stranding of fixed assets including buildings, machinery and equipment and breeding livestock and evaluate land asset value change following dietary transitions. Asset values are based on Farm Accountancy Data Network (FADN) data^20^ (Supplementary Table 1 provides details). In this database, fixed assets represent the existing capital stock, whereas investments in fixed assets are recorded separately as annual capital expenditure. Although ongoing investments may contribute to future stranding risk, they are not yet stranded and are therefore excluded from our analysis.

Other asset categories reported in FADN, such as current assets, financial assets and intangible assets, are not assessed. Financial assets, including short- and medium- to long-term loans, are linked to highly liquid markets which enable risks to be moved^10^. Intangible human and social assets, such as know-how, management practices and community networks, are less vulnerable due to their association with diverse activities^10^ and when monetized, represent less than 2% of the total asset valuation.

We use the Food and Agriculture Biomass Input–Output (FABIO) database (version 2.0), which provides a global series of physical input–output tables for agriculture and food^42^. FABIO v2.0 covers 186 countries and 1 Rest of the World region (*n*^r^*)*, 123 commodities (*n*^s^) and six final demand categories (*n*^y^*)* for 2010–2021. We integrate FABIO with data for 14 farm types (*n*^f^) across EU27 + UK countries provided by the Farm Accountancy Data Network (FADN) data^20^. Asset values for these farm types are proportionally allocated to the *n*^s^ food items using each country’s total output per commodity and a concordance matrix (Supplementary Table 2). Most assets were successfully allocated (land 99.6%, buildings 99.8%, machinery and equipment 99.6% and breeding livestock 100%). FADN data, based on annual EU member state surveys, represent approximately 3.7 million farms across EU27 + UK in 2020, but does not cover fish and seafood assets.

Our input–output analysis approach is inherently static and represents a snapshot of production, trade and efficiency for each year. Our scenario modelling assumes constant technical coefficients and trade structures when projecting into the future. The results should be interpreted as broad estimates of stranding risk under current production systems, providing insight into potential transition exposures rather than precise future outcomes.

We performed a contribution analysis to evaluate the embodied assets across the EU27 + UK food supply chain (that is, agricultural assets accumulated through each supply chain stage). This analysis follows the equation $\mathit{R}^{\mathrm{c}}=\mathbf{\hat{b}}^{\prime}\!\mathit{L}\mathit{Y}$ where ${R}^{{\rm{c}}}$ (*n*^r^*n*^s^ **x** *n*^r^) represents the matrix of embodied impacts for each commodity-region pair. Here $\mathbf{b}^{\prime}$ is a row vector asset intensity (in € t^−1^) calculated by dividing the asset flow $\bf e$ by the total output $\bf x$, as $\mathbf{b}^{\prime}=\mathbf{e}^{\prime} {\hat{\mathbf{x}}}^{-1}$. The Leontief inverse $L$ is given by $L={(I-A)}^{-1}$1, where $I$ is the identity matrix (a matrix with ones on the main diagonal) and $A$ represents the matrix of technical coefficients, all three with the dimensions *n*^r^*n*^s^ **x** *n*^r^*n*^s^. The matrix $Y$ denotes the final demand (*n*^r^*n*^s^ **x** *n*^r^*n*^y^).

To assess potentially stranded assets following a transition to more plant-rich diets, we model the EAT–*Lancet* diet for high- and middle-income countries, considering impacts on EU food consumption, imports and exports. We model three scenarios of ASF intake aligned with the macronutrient intake ranges recommended in the EAT–*Lancet* reference diet^6^ (Supplementary Fig. 1).

1. Moderate ASF scenario, using the upper limit of the ASF intake range (including dairy, beef and lamb, pork, poultry, lard, tallow, eggs and fish) and the lower limit of the range recommended for legumes (including dry beans, lentils and peas, soy food and peanuts) tree nuts and vegetable oils (including palm and unsaturated oils) intake.
2. Low ASF scenario, applying the midpoint for all ASFs, legumes, tree nuts and vegetable oils.
3. Zero ASF scenario, excluding ASF entirely and using the upper range for legumes, tree nuts and vegetable oils.

All dietary scenarios are scaled to an isocaloric intake of 2,500 kcal per person per day, with other plant-based foods adjusted proportionally as needed. Mass-energy conversions were based on FAO Food Balance Sheets^43^. Food waste is factored into both baseline and the dietary scenarios using fixed food-specific fractions^44^. Items not considered by the EAT–*Lancet* recommendation (‘alcohol’ and ‘other’) are excluded (Supplementary Fig. 1). A schematic overview of these methods is provided in Supplementary Fig. 4.

The sensitivity of the asset values is estimated by calculating the minimum and maximum deviations of the 2020 value from the 2014–2019 values, considering the CAP regime for 2014–2020, for each asset type, region and food item. Historical data are adjusted for inflation using the gross domestic product at market prices’ index from Eurostat^45^. As the composition of assets also changes during the dietary transition, we further assessed the sensitivity for each scenario (Fig. 3).

To assess depreciation pathways, we derived the mean depreciation rate from the FADN database using total depreciation allocated to 2020 and depreciable asset values (including fixed assets, permanent crops and quotas)^20^. All data processing and analyses were carried out using Python (version 3.8.8) and RStudio (version 2022.07.2).

### Reporting summary

Further information on research design is available in the Nature Portfolio Reporting Summary linked to this article.

## Supplementary information

Supplementary Information Supplementary Figs. 1–5 and Supplementary Table 1.

Reporting Summary

Supplementary Table 2 Concordance table.

## Source data

Source Data Figs. 1–3 Source data.

## Data Availability

All data used in this study are available in open-access databases. The FABIO database is available via Zenodo at 10.5281/zenodo.2577067 (ref. ^42^), and the FADN Public Database is available via the agridata platform of the European Commission (https://agridata.ec.europa.eu/extensions/FADNPublicDatabase/FADNPublicDatabase.html). Source data are provided with this paper.

## Code availability

Example code of the performed analyses is available via GitHub at https://github.com/fineprint-global/fabio.
