# Supplementary material for: Stranded assets in European agriculture during food system transformations
Source: Nat Food. 2026 Jan 19;7(1):38–44. doi: 10.1038/s43016-025-01283-z (PMC12851926; doi:10.1038/s43016-025-01283-z)
Supplement: Supplementary file 1 — Supplementary Figs. 1–5 and Supplementary Table 1. [file 43016_2025_1283_MOESM1_ESM.pdf]

nature food

Supplementary information

https://doi.org/10.1038/s43016-025-01283-z

Stranded assets in European agriculture during food system transformations

In the format provided by the authors and unedited

Supplementary information: Stranded assets in European agriculture during food system transformations

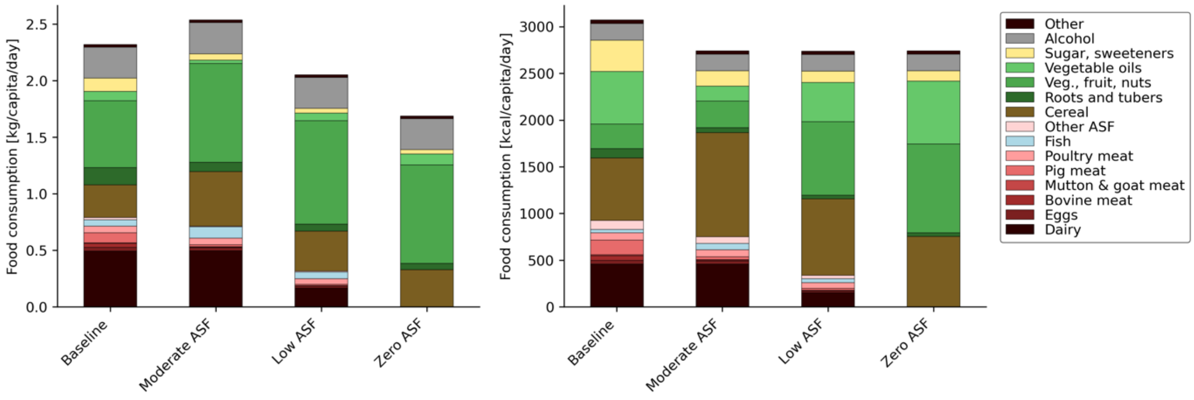

Fig. 1. EU27+UK food intake and dietary changes, excluding food waste (left: mass, right: energy).

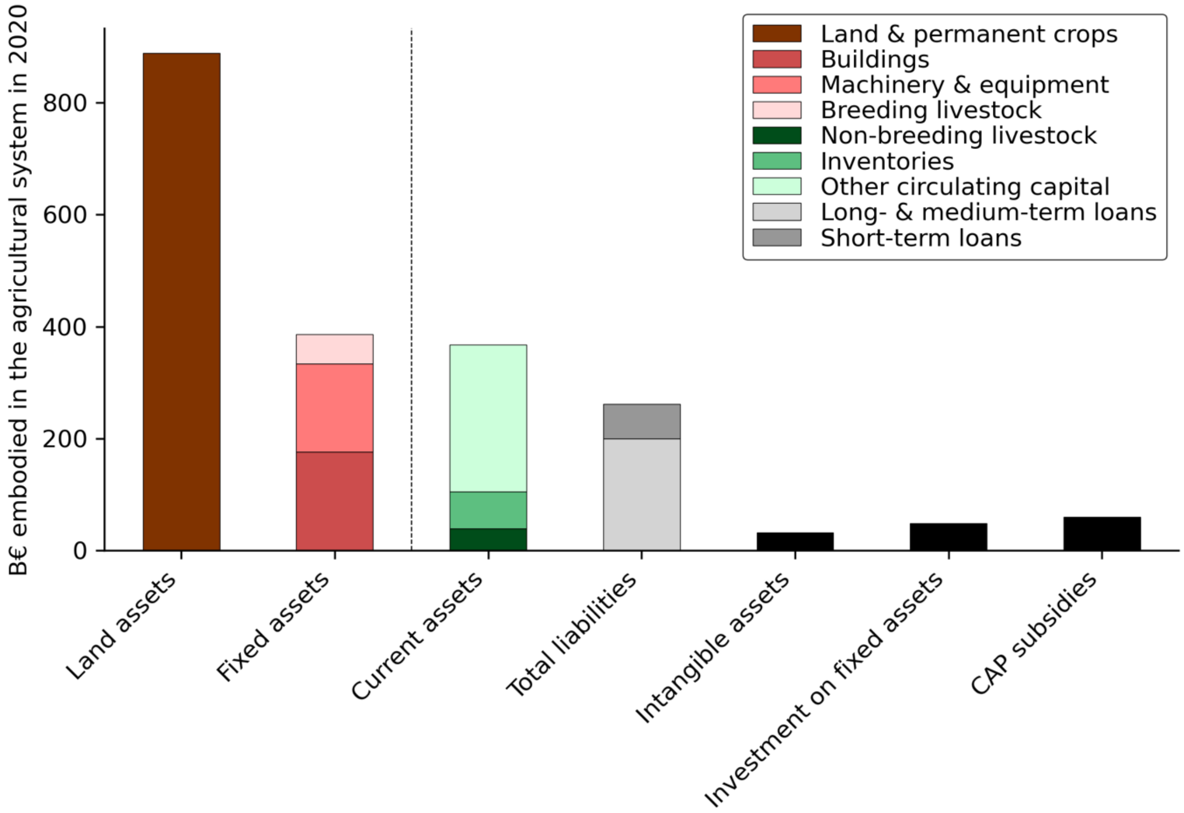

Fig. 2. Overview of asset types within the EU27+UK agricultural system in 2020. Land assets include land value and permanent crops (e.g., orchards, vineyards). Fixed assets are divided into buildings, machinery & equipment, and breeding-livestock. Current assets include non-breeding livestock, inventories (stocks of products owned by the farm for input use or sale, whether produced or purchased), and other current assets (cash, business receivables, and assets easily sold or payable within a year). Total liabilities, representing farm debt, include short-term and long- to medium-term loans. Intangible assets are either tradable (quotas, rights) or non-tradable (software, licences). CAP subsidies include the full CAP budget for the EU27+UK. For assets linked to food production only, see Fig. 1.

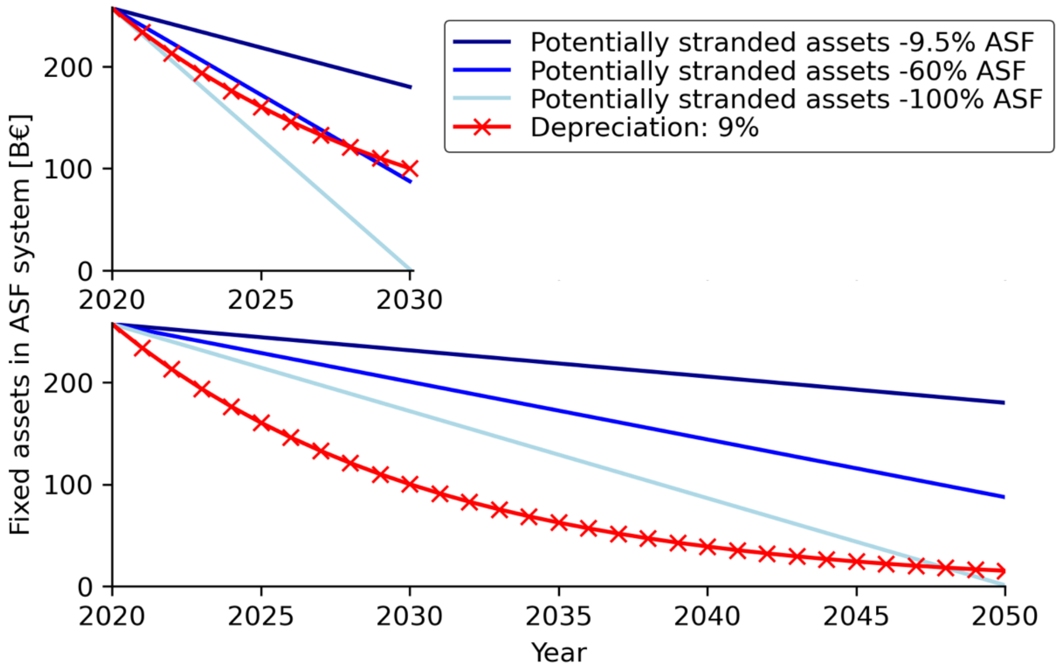

Fig. 3. Linear transition to ASF reductions and depreciation of fixed assets over a time span of 10 years (upper plot) and 30 years (lower plot). Transitions are modelled from a 2020 baseline, based on the most recent year for which data are available.

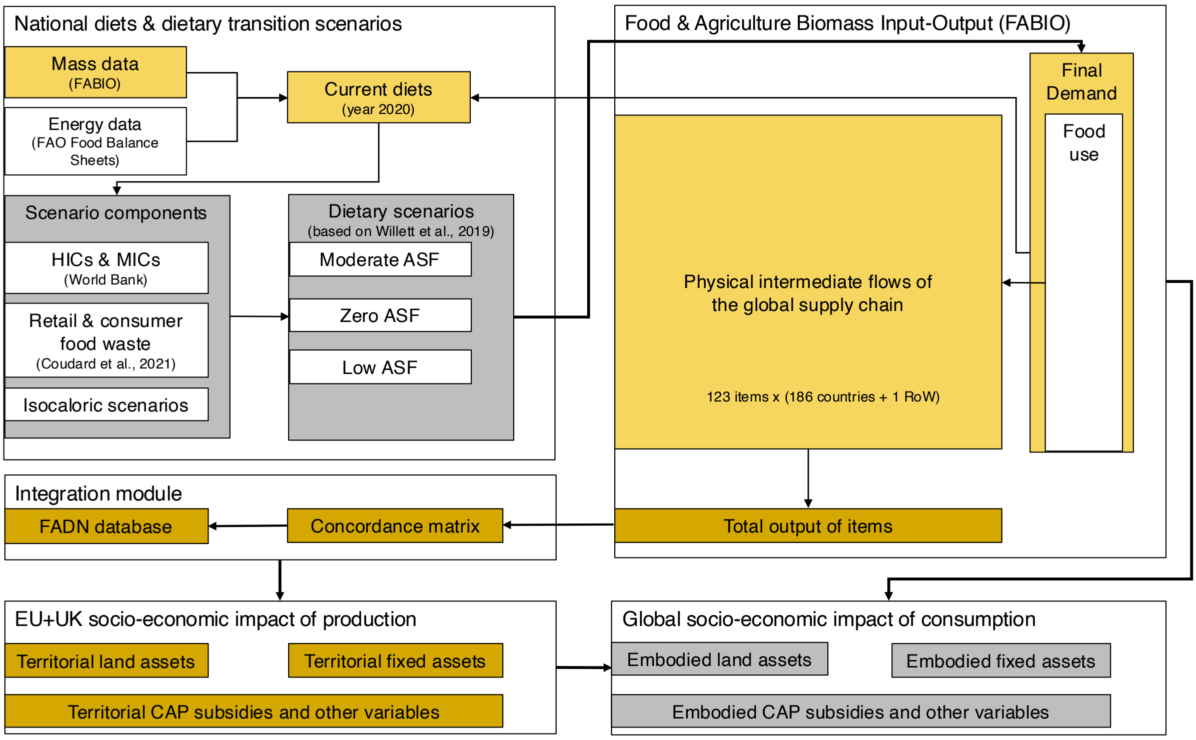

Fig. 4. Methods schematic of the dietary scenario construction, the FADN data reorganization and its integration with FABIO to analyse the socio-economic impact of food consumption.

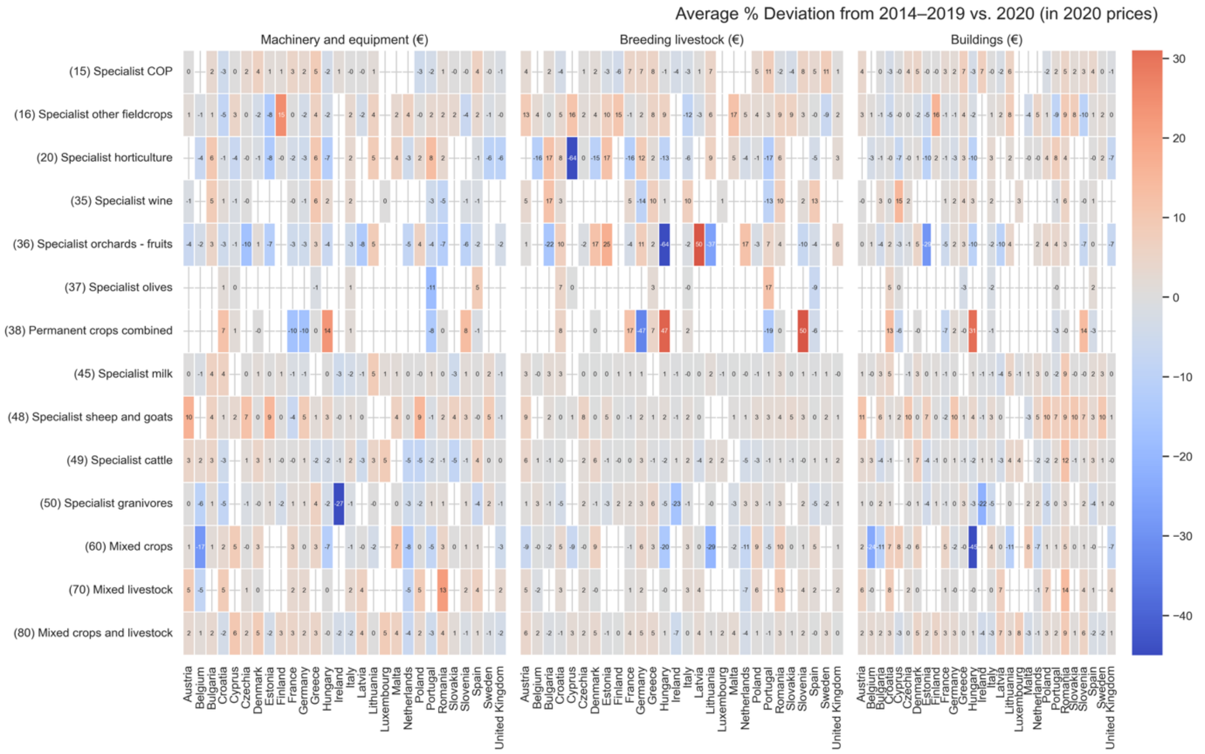

Fig. 5. Sensitivity of each FADN data point represented as the deviation of the 2020 value from the average 2014-2019 values in 2020 prices.

Table 1. Overview of FADN data

Category:	Subcategory:	Description:	Collected as:
Land asset	Land	Agricultural land, permanent crops, improvements to land, quotas and other prescribed rights (including acquisition costs) and forest land<br><br>Agricultural land owned by the holding including land improvements (e.g., fencing, drainage, fixed irrigation equipment) belonging to the holder.<br><br>Forest land included stranding timber in owner occupation included in the agricultural holding	“biological assets – plants” + “agricultural land” + “land improvements” + “forest land including stranded timber” + “intangible assets, tradable”
Fixed assets	Buildings	Farm buildings belonging to the holder whatever the type of occupancy of the land	
	Machinery and equipment	Machinery and equipment including tractors, motor cultivators, lorries, vans, cars, major and minor farming equipment.	
	Breeding livestock	Breeding livestock including breeding heifers, dairy cows, buffalo dairy cows, non-dairy cows, breeding female sheep, breeding female goats, breeding sows	
Depreciation		Depreciation of capital assets. Concerns plantations of permanent crops (biological assets, estimated), farm buildings and fixed equipment, land improvements, machinery and equipment and forest plantations. There is no depreciation of land and circulating capital (= short-term assets that can be easily converted to cash within the accounting year). There is depreciation of non-tradable intangible assets.	Recorded in the table as “depreciation of the current year”<br><br>Accumulated depreciation is used to valuate assets at closing valuation
