# Supplementary material for: Stranded assets in European agriculture during food system transformations
Source: Nat Food. 2026 Jan 19;7(1):38–44. doi: 10.1038/s43016-025-01283-z (PMC12851926; doi:10.1038/s43016-025-01283-z)
Supplement: Supplementary file 2 — Reporting Summary [file 43016_2025_1283_MOESM2_ESM.pdf]

Corresponding author(s): A.J. Kortleve & P. Behrens

Last updated by author(s): 03/12/2025

Reporting Summary

Nature Portfolio wishes to improve the reproducibility of the work that we publish. This form provides structure for consistency and transparency in reporting. For further information on Nature Portfolio policies, see our Editorial Policies and the Editorial Policy Checklist.

Statistics

For all statistical analyses, confirm that the following items are present in the figure legend, table legend, main text, or Methods section.

n/a	Confirmed	
☐	☒	The exact sample size (*n*) for each experimental group/condition, given as a discrete number and unit of measurement
☒	☐	A statement on whether measurements were taken from distinct samples or whether the same sample was measured repeatedly
☒	☐	The statistical test(s) used AND whether they are one- or two-sided<br>*Only common tests should be described solely by name; describe more complex techniques in the Methods section.*
☒	☐	A description of all covariates tested
☒	☐	A description of any assumptions or corrections, such as tests of normality and adjustment for multiple comparisons
☒	☐	A full description of the statistical parameters including central tendency (e.g. means) or other basic estimates (e.g. regression coefficient) AND variation (e.g. standard deviation) or associated estimates of uncertainty (e.g. confidence intervals)
☒	☐	For null hypothesis testing, the test statistic (e.g. *F*, *t*, *r*) with confidence intervals, effect sizes, degrees of freedom and *P* value noted<br>*Give P values as exact values whenever suitable.*
☒	☐	For Bayesian analysis, information on the choice of priors and Markov chain Monte Carlo settings
☒	☐	For hierarchical and complex designs, identification of the appropriate level for tests and full reporting of outcomes
☒	☐	Estimates of effect sizes (e.g. Cohen's *d*, Pearson's *r*), indicating how they were calculated

Our web collection on statistics for biologists contains articles on many of the points above.

Software and code

Policy information about availability of computer code

Data collection	Data processing was carried out using Python version 3.8.8 and Rstudio version 2022.07.2.
Data analysis	The data consist of a physical multi-regional input-output model which was extended with socio-economic data. The extended model was analyzed using standing input-output calculations, which are described in the methodology of the manuscript. The code used for this study is available upon request, however, example code of similar analyses is provided by the FABIO group on: https://github.com/fineprint-global/fabio.

For manuscripts utilizing custom algorithms or software that are central to the research but not yet described in published literature, software must be made available to editors and reviewers. We strongly encourage code deposition in a community repository (e.g. GitHub). See the Nature Portfolio guidelines for submitting code & software for further information.

Data

Policy information about availability of data

All manuscripts must include a data availability statement. This statement should provide the following information, where applicable:

- Accession codes, unique identifiers, or web links for publicly available datasets
- A description of any restrictions on data availability
- For clinical datasets or third party data, please ensure that the statement adheres to our policy

All data used in this study are available in open-access databases. The FABIO database in available via Zenodo (https://doi.org/10.5281/zenodo.2577067) and the

Farm Accountancy Data Network (FADN) Public Database in available via the agridata platform of the European Commission (https://agridata.ec.europa.eu/extensions/FADNPublicDatabase/FADNPublicDatabase.html). Full MRIO table results are available upon request. These can also be replicated using the public databases in combination with the information provided in the methods section and supplementary information.

Human research participants

Policy information about studies involving human research participants and Sex and Gender in Research.

Reporting on sex and gender	n/a
Population characteristics	n/a
Recruitment	n/a
Ethics oversight	n/a

Note that full information on the approval of the study protocol must also be provided in the manuscript.

Field-specific reporting

Please select the one below that is the best fit for your research. If you are not sure, read the appropriate sections before making your selection.

☐ Life sciences ☐ Behavioural & social sciences ☒ Ecological, evolutionary & environmental sciences

For a reference copy of the document with all sections, see nature.com/documents/nr-reporting-summary-flat.pdf

Ecological, evolutionary & environmental sciences study design

All studies must disclose on these points even when the disclosure is negative.

Study description	The physical multi-regional input-output database (FABIO) was extended by integrating socio-economic data (land assets, fixed assets, current assets, total liabilities, intangible assets, investment on fixed assets, and CAP subsidies) for all EU+UK countries. This integration was achieved through a concordance table provided in the supplementary information. It enabled tracking of socio-economic extensions through the global agricultural supply chain and modeling the impact of dietary transition scenarios on these extensions.
Research sample	n/a
Sampling strategy	n/a
Data collection	n/a
Timing and spatial scale	Year 2020 and global scale. The years 2014-2019 are used for the sensitivity analysis.
Data exclusions	n/a
Reproducibility	n/a
Randomization	n/a
Blinding	n/a

Did the study involve field work? ☐ Yes ☒ No

Reporting for specific materials, systems and methods

We require information from authors about some types of materials, experimental systems and methods used in many studies. Here, indicate whether each material, system or method listed is relevant to your study. If you are not sure if a list item applies to your research, read the appropriate section before selecting a response.

Materials & experimental systems

n/a	Involved in the study
☒	☐ Antibodies
☒	☐ Eukaryotic cell lines
☒	☐ Palaeontology and archaeology
☒	☐ Animals and other organisms
☒	☐ Clinical data
☒	☐ Dual use research of concern

Methods

n/a	Involved in the study
☒	☐ ChIP-seq
☒	☐ Flow cytometry
☒	☐ MRI-based neuroimaging
